# Thermotropic and lyotropic behaviour of new liquid-crystalline materials with different hydrophilic groups: synthesis and mesomorphic properties

**DOI:** 10.3762/bjoc.9.45

**Published:** 2013-02-25

**Authors:** Alexej Bubnov, Miroslav Kašpar, Věra Hamplová, Ute Dawin, Frank Giesselmann

**Affiliations:** 1 Institute of Physics, Academy of Sciences of the Czech Republic, Na Slovance 2, 182 21 Prague, Czech Republic; 2 Institute of Physical Chemistry, University of Stuttgart, Pfaffenwaldring 55, 70569 Stuttgart, Germany

**Keywords:** contact cell preparation, hydrophilic chain, lamellar phase, lyotropic liquid crystal, thermotropic liquid crystal

## Abstract

Several new calamitic liquid-crystalline (LC) materials with flexible hydrophilic chains, namely either hydroxy groups or ethylene glycol units, or both types together, have been synthesized in order to look for new functional LC materials exhibiting both, thermotropic and lyotropic behaviour. Such materials are of high potential interest for challenging issues such as the self-organization of carbon nanotubes or various nanoparticles. Thermotropic mesomorphic properties have been studied by using polarizing optical microscopy, differential scanning calorimetry and X-ray scattering. Four of these nonchiral and chiral materials exhibit nematic and chiral nematic phases, respectively. For some molecular structures, smectic phases have also been detected. A contact sample of one of the prepared compounds with diethylene glycol clearly shows the lyotropic behaviour; namely a lamellar phase was observed. The relationship between the molecular structure and mesomorphic properties of these new LCs with hydrophilic chains is discussed.

## Introduction

All materials showing liquid-crystalline (LC) behaviour belong to two general classes: lyotropic materials, in which fluid anisotropy results from interactions between anisotropic aggregates of amphiphilic molecules; and thermotropic materials, in which the orientational order arises from interactions among partially rigid anisotropic molecules. Amphiphilic compounds can form a variety of thermotropic liquid-crystalline phases as a function of temperature by themselves, and lyotropic liquid-crystalline phases upon addition of some solvent, such as water or diethylene glycol [1]. Thermotropic liquid-crystalline systems composed of amphiphilic molecules can exhibit smectic phases with layered structure [2] or nematic (eventually cholesteric) phases. Lyotropic systems usually form a lamellar liquid-crystalline mesophase, i.e., a lyotropic analogue of the thermotropic orthogonal smectic A (SmA) phase [3], and more rarely nematic, columnar and cubic mesophases [4–5].

Combination of thermotropic and lyotropic properties for materials with definite molecular structure has been intensively studied so far [6–16] and is of high importance in particular for developing new types of functional LC materials [15,17–18]. Series of alkyl glucosides and related materials usually possess thermotropic mesophases [16] as well as lyotropic columnar phases in water solution [3]. The thermotropic SmA phase and hexatic/cubic/lamellar lyotropic phases have been detected for a series of 4-alkoxyphenyl β-D-glucopyranosides [5]. LC compounds in which a hydrophilic polyethyleneimine chain with hydroxy side-groups is attached to the azobenzene mesogenic core through the alkylene chain have showed a thermotropic SmA phase, lyotropic lamellar phase, and a lyotropic analogue of the tilted smectic C phase [2]. Due to microsegregation of the hydrophilic regions from aromatic segments in the absence of flexible alkyl chains, the thermotropic and lyotropic smectic and columnar phases have been detected for 4-benzyloxy-4’-(2,3-dihydroxypropyloxy)biphenyls with lateral methyl substituents [19]. Several silver-containing thermotropic liquid-crystalline materials based on bis(stilbazole) silver(I) cation in association with the amphiphilic counter-ion lauryl sulfate exhibit lyotropic and thermotropic liquid-crystalline behaviour [20]. A series of monoalkyl glycosides possesses a very stable thermotropic SmA phase as well as lyotropic lamellar, cubic and even hexagonal phases depending on the amount of the added water used as a solvent [4]. Intensive studies have been done on long-alkyl-chain glycopyranosides with monosaccaride [21] and disaccharide [22] and compounds with pH-sensitive [23] head groups. For these types of compounds the thermotropic SmA and cubic phases have been detected. In addition, rich lyotropic polymorphism has also been found, namely cubic phases and lamellar phases as well as the lyotropic cholesteric phase [21–23]. An effort to identify the role of the hydroxy group in the mesomorphic behaviour of alkyl glycosides has been made recently [24]. The methyl 6-O-(*n*-acyl)-α-D-glucopyranosides, with chain lengths between dodecanoyl and hexadecanoyl exhibit a monotropic SmA phase (i.e., occurring explicitly on cooling) only [25]. Even though a lot has already been done, many open questions still remain.

The objective of this work is to contribute to a better understanding of the chemical-structure–physical-property relationship for materials forming both, thermotropic and lyotropic mesophases. This understanding is of high potential interest for challenging issues such as the organization of carbon nanotubes [17] or various nanoparticles [18] in a liquid-crystalline matrix. To reach the goal, several new multifunctional liquid-crystalline materials possessing either ethylene glycol units (denoted as **TL1** and **TL2**) or a different number of hydroxy groups (denoted as **TL3** and **TL4** [26–28]) or both types of these hydrophilic groups together and an additional photosensitive azo group in the molecular core (denoted as **TL5**) have been designed, synthesized and studied (see Table 1 for the chemical structures). Compound **TL4** with a decyloxy end group and a hexyl spacer was described in [28] along with a similar compound having a hexyloxy end group. A compound that carries a hexyloxy group and a pentyl spacer has been studied recently [27]; mesomorphic behaviour of this material was very similar to that of **TL4**. Corresponding mesophases N* and SmC* were also observed for the chiral compound in [26] having the same molecular core as **TL4** but a branched chiral alkoxy chain instead of the decyloxy group used for **TL4**. The relation between the molecular structure and mesomorphic properties of these new multifunctional compounds with different structures is discussed.

**Table 1:** Chemical formulae of compounds and used abbreviations.

Notation	Chemical formula

**TL1**	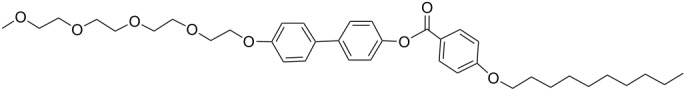 4'-(2,5,8,11-tetraoxatridecan-13-yloxy)biphenyl-4-yl 4-(decyloxy)benzoate
**TL2**	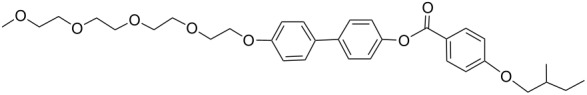 4'-(2,5,8,11-tetraoxatridecan-13-yloxy)biphenyl-4-yl 4-(2-methylbutoxy)benzoate
**TL3**	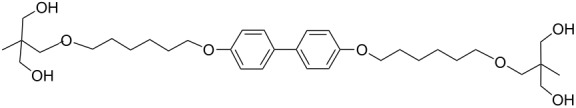 2-(1-(6-(4'-(6-(3-hydroxy-2-(hydroxymethyl)-2-methylpropoxy)hexyloxy)biphenyl-4-yloxy)hexyloxy)methyl)-2-methylpropane-1,3-diol
**TL4**	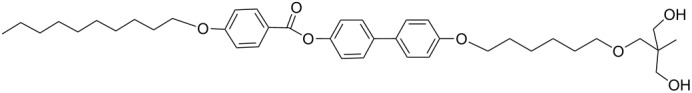 4'-(6-(3-hydroxy-2-(hydroxymethyl)-2-methylpropoxy)hexyloxy)biphenyl-4-yl 4-(decyloxy)benzoate
**TL5**	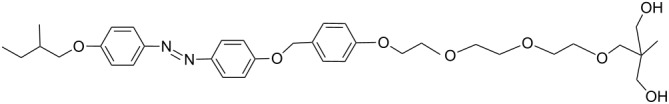 (*E*)-2-methyl-2-((2-(2-(2-(4-((4-((4-(2-methylbutoxy)phenyl)diazenyl)phenoxy)methyl)phenoxy)ethoxy)ethoxy)ethoxy)methyl)propane-1,3-diol

## Results and Discussion

### Thermotropic behaviour

#### Mesomorphic properties

For the studied materials, sequences of phases were determined by characteristic textures and their changes were observed in a polarizing optical microscope (POM). The phase-transition temperatures and transition enthalpies were evaluated from differential scanning calorimetry (DSC) measurements. Thermotropic liquid-crystalline properties of all compounds under study are summarized in Table 2.

**Table 2:** Sequence of phases and phase-transition temperatures, *T* (°C), measured on cooling (5 K min^−1^); melting points, mp (°C), measured on heating (5 K min^−1^) and phase-transition enthalpies, Δ*H* [Jg^−1^], obtained by DSC for the studied compounds.^a^

	mp	phase	*T*/Δ*H*	phase	*T*/Δ*H*	phase	*T*/Δ*H*	phase	*T*/Δ*H*	phase	*T*/Δ*H*	Iso

**TL1** (nonchiral)	70 [+19.0]	Cr2	54 [−18.1]	Cr1	90 [−16.3]	SmC	102 [−3.5]	N	115 [−2.0]	–		●
**TL2** (chiral)	80 [+39.9]	Cr2	70 [−36.0]	–		–		N*	103 [−0.7]	BPII	104 [msc]	●
**TL3** (nonchiral)	74 [+39.9]	Cr2	69 [−43.0]	Cr1	117 [−67.6]	–		–		–		●
**TL4** (nonchiral)	76 [+82.9]	Cr2	58 [−45.1]	Cr1	109 [−62.9]	SmC	147 [−17.6]	N	157 [−9.6]	–		●
**TL5** (chiral)	95 [+7.6]	Cr2	86 [−5.6]	–		SmC*	113 [−0.7]	N*	120 [−16.5]	–		●

^a^(“●” - the phase exists; “–” - the phase does not exist; “[msc]” - determined by observations in POM only).

On cooling from the isotropic state, **TL1** and **TL4**, with quite a different structure of the molecular core and aliphatic chains (see Table 1 for the difference in molecule structure), possess the nematic phase. The tilted smectic phase (SmC) being partially monotropic in character (i.e., the high temperature part of the phase is enantiotropic) has been observed on cooling below the nematic phase. For **TL4**, the temperature of the Iso–N phase transition (157 °C) is found to be pronouncedly higher than that observed for **TL1**. Below the SmC phase a crystal modification (denoted as Cr1 phase) has been detected for **TL4** and **TL1**. For **TL3** no liquid-crystalline behaviour has been detected: only a modification of a crystalline phase (denoted as Cr1) exists within quite a broad temperature range.

**Figure 1:**
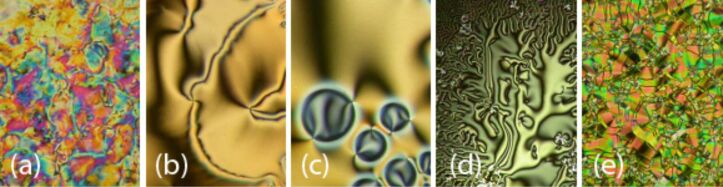
Microphotographs of the textures obtained in the polarized optical microscope on planar samples (PS) and free-standing films (FSF) as indicated: (a) The nematic phase at 110 °C for **TL1** (PS); (b) the nematic phase at 108 °C for **TL1** (FSF); (c) the N–SmC phase transition at about 102 °C for **TL1** (FSF); (d) the chiral nematic N* phase at 111 °C for **TL5** (PS); (e) “broken fans” of the SmC* phase at 106 °C for **TL5** (PS). (Width of the photos is about 300 μm).

In Figure 1 microphotographs of textures for **TL1** taken under different temperature and alignment conditions are presented, namely the marbled texture of the nematic phase obtained at 110 °C on a planar cell (Figure 1a), the schlieren texture of the nematic phase obtained at 108 °C on free-standing films (FSF) (Figure 1b), and the N–SmC phase transition obtained at about 102 °C on FSF (Figure 1c).

**Figure 2:**
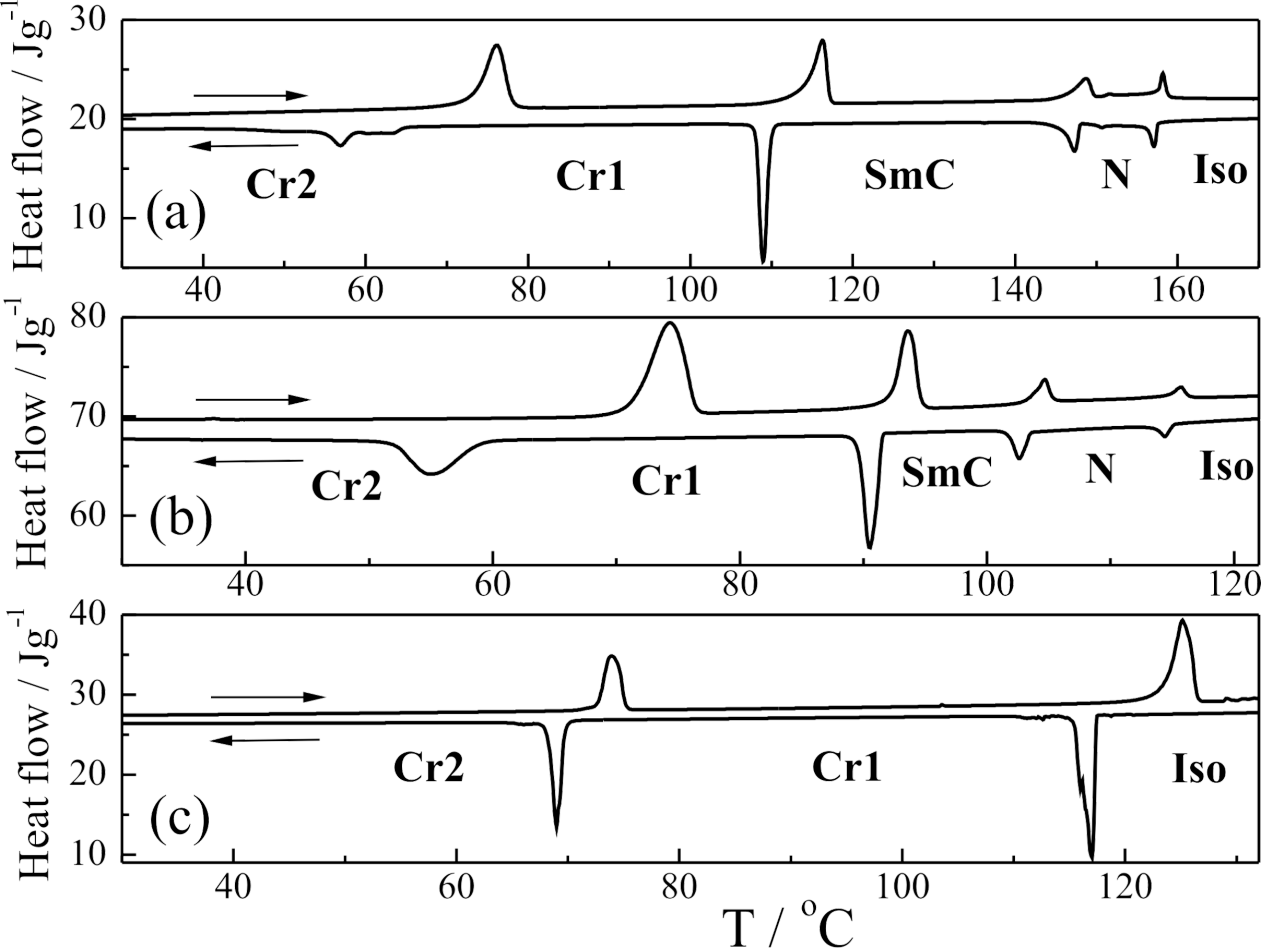
DSC plot on heating/cooling runs (indicated by horizontal arrows) for indicated nonchiral compounds: **TL4** (a), **TL1** (b) and **TL3** (c). The detected mesophases are indicated.

**Figure 3:**
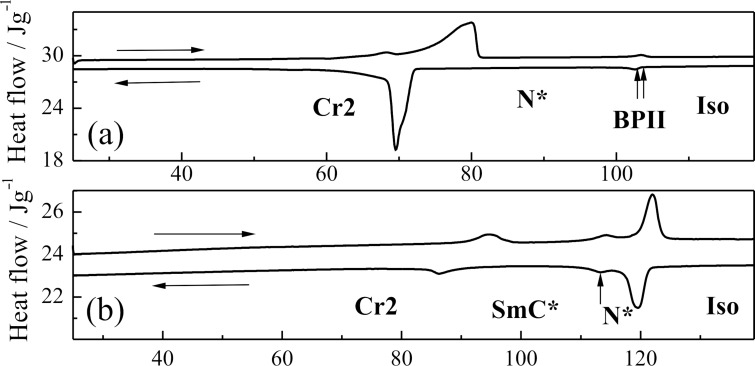
DSC plot on heating/cooling runs (indicated by horizontal arrows) for indicated chiral compounds: **TL2** (a) and **TL5** (b). The detected mesophases are indicated.

DSC plot on heating/cooling runs for indicated nonchiral **TL1, TL3** and **TL4** and for chiral **TL2** and **TL5** are presented in Figure 2 and Figure 3, respectively. The blue phase (BPII) and the cholesteric phase (N*) have been detected for the chiral **TL2**. The peak corresponding to the Iso-BPII phase transition has not been detected by DSC and the temperature of this phase transition has been taken from observations obtained by POM (Table 2). The type of the blue phase (BPII) has been determined by the characteristic platelet texture (similar to that presented in [29]) observed on planar samples by POM. Chiral **TL5** with an azo group in the molecule core possesses the chiral nematic phase and the tilted ferroelectric smectic C* phase. Ferroelectric electro-optic switching has been clearly detected in the SmC* phase but due to the monotropic (supercooled) character of the phase it was not possible to measure and study the spontaneous quantities in detail, namely the spontaneous polarization and tilt angle. For this compound the microphotographs of textures obtained in POM are shown on Figure 1d and Figure 1e for the chiral nematic N* phase at 111 °C (planar sample) and for the ferroelectric tilted SmC* phase (broken fans texture) at 106 °C, respectively.

#### X-ray scattering

**Figure 4:**
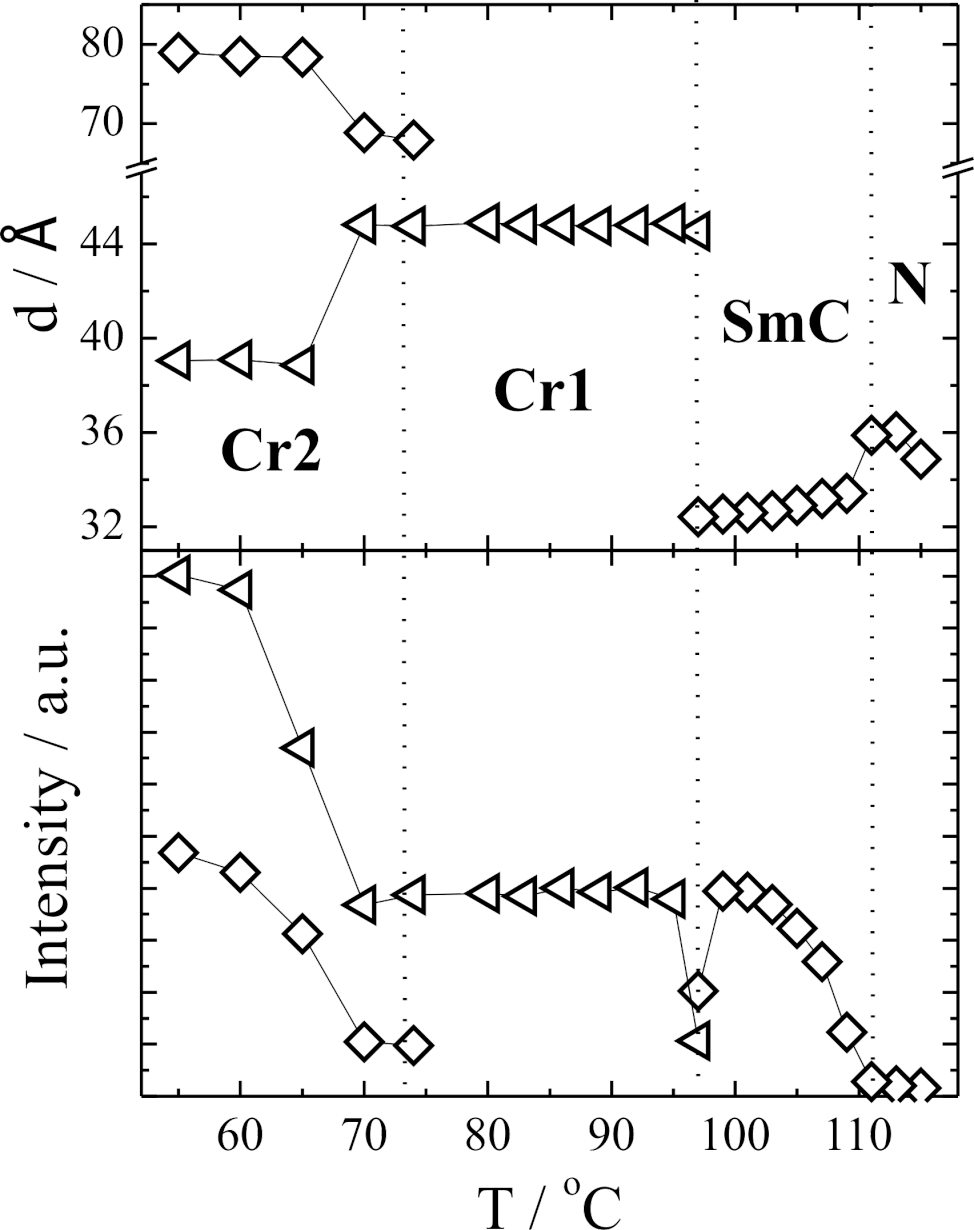
Temperature dependence of the layer spacing *d*, and intensity of the scattered X-ray beam measured at small angles in the nematic, SmC and crystalline phases for **TL1**. Values of the layer spacing obtained in the nematic phase correspond to the length of the molecule.

The structures of the mesophases formed in the studied compounds have been checked by X-ray scattering. As an example, the temperature dependence of the smectic layer spacing *d*, for **TL1** is shown in Figure 4. The MOPAC/AM1 model has been used to calculate the length of **TL1** in the energy-optimized conformation, which gives 41 Å for the most extended conformer. In X-ray data taken at small scattering angles in the nematic phase, a peak of quite a low intensity corresponding to this scattering usually approximately matches the molecular length and could also give evidence of the pretransitional smectic order. For **TL1**, these values are substantially lower than the calculated length of the most extended conformer. This fact gives evidence for the bent-like conformation of the molecule, i.e., the molecule core is rigid but the flexible chains are bent and not extended at all. The increase of scattered X-ray signal intensity on cooling also confirms the increase of the order with temperature decrease (Figure 4). In the SmC phase, the decrease of the *d* values is due to the increase of the molecule tilt angle on cooling. The width of the mesophases and hence the phase-transition temperatures detected from the X-ray analysis are slightly shifted with respect to those determined from the DSC (Table 2) due to a different cooling rate.

### Lyotropic behaviour

In this section preliminary studies of lyotropic behaviour by means of contact preparation are presented.

#### Contact preparation

**Figure 5:**
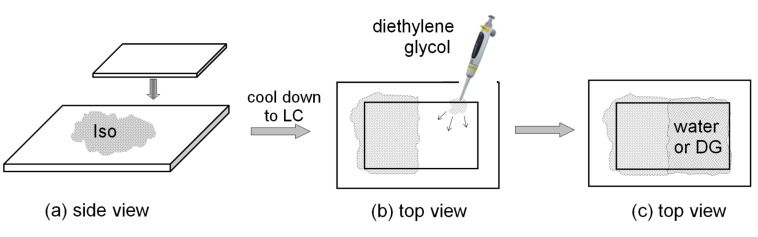
Schematic contact preparation used for detection and study of lyotropic behaviour.

The cells for the contact preparation were prepared as schematically presented in Figure 5. On a glass slide an amount of the material (several milligrams) was melted into the isotropic phase (Figure 5a). The liquid was covered by another glass slide in such a way that only half of the area between the slides was filled by the LC material (Figure 5b). At the LC–air interface a drop of water or diethylene glycol (DG) was placed close to the cover glass on one side only, in order to ensure that water or DG fills the air gap at the LC border without leaving air bubbles (Figure 5c). The thickness of the cell is not fixed, but preferably is within 10–50 μm, which can be detected by interferometry. Contact samples were studied by using POM up to the evaporation temperature of the solvent (which is not desirable as the solvent condensates on the lenses of the POM objective). The cells described above are easy to prepare and very convenient for primary identification of the lyotropic behaviour if any exists. Sealed samples with well-defined thickness (fixed by spherical glass spacers, Figure 5a) were also used. A special silicon glue stable up to 250 °C was used for sealing (Figure 5c) preventing evaporation of the solvent (usually water or DG).

#### Mesomorphic lyotropic behaviour

**Figure 6:**
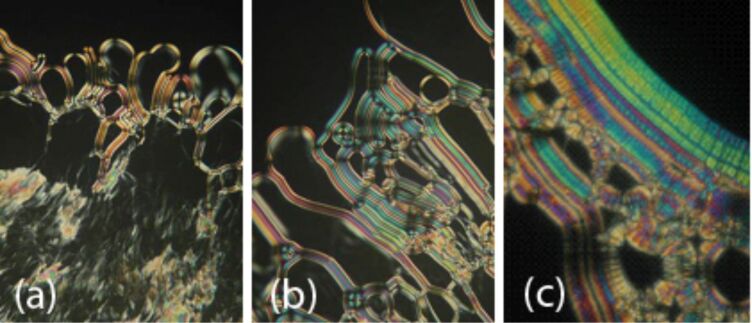
Microphotographs of the contact preparation of **TL4** with diethylene glycol (DG): (a) texture of the lamellar phase (on top) at about 109.0 °C (width of the photo is approx. 350 μm); (b) and (c) close-up texture of the lyotropic area: myelinic streaks appear within a few seconds and remain stable; black area on top of the microphotographs corresponds to homeotropic orientation of the lamellar phase or isotropic liquid regions of mainly DG (width of the photo is about 150 μm).

**TL1–TL5** were tested for lyotropic behaviour by using the contact-cell preparation technique. For our studies, water and/or diethylene glycol were used as the solvent. However, after careful testing of all the materials only **TL4** clearly shows the lyotropic phase in contact with diethylene glycol starting at the temperature of 109 °C, which is lower than the thermotropic crystal–LC phase-transition temperature (*T*_cryst–LC_ = 113 °C). On heating, the solvent visibly penetrates into the liquid-crystalline area of **TL4** material indicating the solubility. Figure 6a shows the overview of the contact area. Figure 6b,c provides a microphotograph of a close-up of the lyotropic area. The myelinic streaks can be taken as clear evidence for the existence of the lamellar phase. However, more detailed studies of the phase diagram are necessary (in progress now; will be presented elsewhere) in order to get more quantitative results on the lyotropic behaviour of **TL4**.

### Discussion of the molecular structure – mesomorphic property relationship

It is quite obvious that the structure of the molecular core as well as the presence of additional terminal hydroxy groups or alkyl chains should significantly influence the mesomorphic properties of LC materials. Recently, two series of chiral liquid-crystalline materials (similar to those presented in this work) with chiral lactate group or lactic acid derivative have been synthesized and studied [30–32]. These materials (with the same molecular core and nonchiral chain as **TL4**) possess different thermotropic LC phases depending on the chiral chain structure. However, the temperatures of the phase transitions are remarkably lower for these chiral materials than are those for **TL4**, as the introduction of two hydroxy groups instead of the chiral chain usually results in an increase of the clearing point.

Mesomorphic properties of **TL5** with two hydroxy groups can be compared to those of the chiral compounds with terminal acrylic group in the nonchiral chain prepared as monomers for side-chain polyacrylates [33–34]. All these compounds clearly possess the LC behaviour and exhibit the chiral nematic phase and also the chiral smectic phases. No pronounced difference in clearing point has been found in cases where the molecule core is the same as for **TL5** but the nonchiral chain with a double bond is replaced by that with two hydroxy groups. However, a minor increase in the melting point (about 10 K) has been observed for **TL5** with respect to that of the chiral acrylates [34].

Quite remarkable observations can be made while comparing the mesomorphic properties of **TL2** with those of a compound possessing the same molecular core and chiral centre but differing in the nonchiral chain, i.e., an ethylene glycol unit has been used instead of the alkyl chain [35]. The presence of ethylene glycol units destroys the smectic order. Hence, the chiral nematic phase is favoured by **TL2** instead of the tilted ferroelectric SmC* phase observed for the compound with the nonchiral alkyl chain. However, the presence of the ethylene glycol units suppresses the clearing point by more than 30 K for **TL2** compared to that for a material with a typical alkyl chain [35].

While searching for new LCs exhibiting both the thermotropic and lyotropic behaviour, materials with a hydrophilic ethylene glycol chain (**TL1** and **TL2**) instead of the frequently used hydrophobic alkyl terminal chains have been synthesized. However, our studies by contact preparation show that no lyotropic behaviour is present. Moreover, the symmetric, strongly hydrophilic material with diol moieties at the end of both alkyl chains (**TL3**) also does not possess the lyotropic behaviour, and even the thermotropic one is absent. A combination of the ethylene glycole group together with a terminal diol moiety on both alkyl chains (**TL5**) has been also found useless for the presence of simultaneous thermotropic and lyotropic behaviour. Only asymmetric **TL4** with a diol moiety terminating one alkyl chain possesses both, thermotropic and lyotropic phases.

## Conclusion

Several new liquid-crystalline materials possessing either two or four hydroxy groups or ethylene glycol units, or both types together, have been synthesized and studied with the aim to look for new functional liquid-crystalline materials exhibiting the thermotropic and lyotropic behaviour. Most of these new nonchiral and chiral materials exhibit the thermotropic nematic and chiral nematic phases, respectively. Smectic phases have been detected for the studied materials with the exception of nonchiral **TL3** and chiral **TL2**. **TL3** possesses no liquid-crystalline phases but only crystalline ones. Lyotropic behaviour has been tested for all LC materials by using a contact preparation technique, which has been described in detail. A contact sample of **TL4** with diethylene glycol clearly shows the lyotropic behaviour, namely a lamellar phase was detected. While looking for advanced functional LC materials, such a type of molecular structure seems to be promising for further studies, which are in progress now and will be presented elsewhere.

## Experimental

### Synthesis

The synthetic procedure for the liquid-crystalline diol with a 1,3-propandiol group connected by a flexible spacer to the mesogenic part of the molecule and with two hydroxy groups (4'-(6-(3-hydroxy-2-(hydroxymethyl)-2-methylpropoxy)hexyloxy)biphenyl-4-yl 4-(decyloxy)benzoate), denoted as **TL4**, has been presented in [28]. The synthesis of the other compounds is presented in this section. Structures of the intermediate and final products were confirmed by ^1^H nuclear magnetic resonance spectroscopy by using a 300 MHz Varian spectrometer in solutions with CDCl_3_ or dimethylsulfoxide (DMSO) with tetramethylsilane as an internal standard. The chemical purity of the compounds was checked by high performance liquid chromatography, which was carried out with an Ecom HPLC chromatograph by using a silica-gel column (Separon 7 μm, 3 × 150, Tessek) with a 98/2 mixture of toluene and methanol as eluent (typical flow rate 1 mL/min, retention times for compounds **TL1**–**TL5** being from 3 min to 12 min). Detection of the eluting products was done by UV–vis detector (λ = 290 nm). For **TL2** and **TL5** the chiral centres are in (*S*)-configuration. The chemical purity of all synthesised compounds was found within 99.5–99.9%.

#### Preparation of **TL1** and **TL2**

**Scheme 1:**
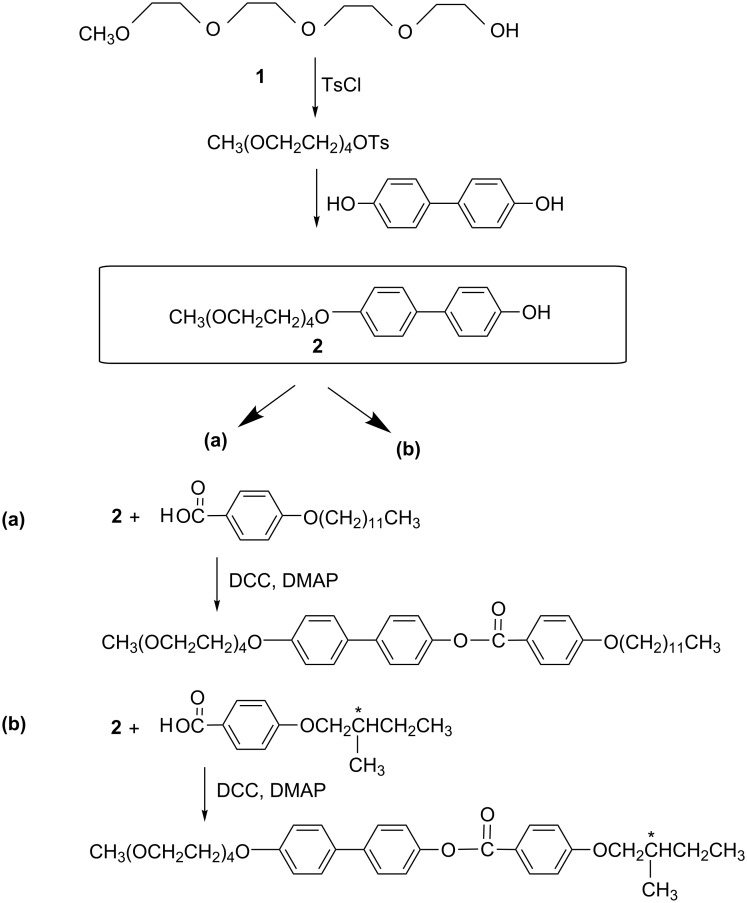
General procedure for the synthesis of (a) nonchiral 4'-(2,5,8,11-tetraoxatridecan-13-yloxy)biphenyl-4-yl 4-(decyloxy)benzoate (**TL1)** and (b) chiral 4'-(2,5,8,11-tetraoxatridecan-13-yloxy)biphenyl-4-yl 4-(2-methylbutoxy)benzoate (**TL2**) with ethylene glycol units.

The general procedure for the synthesis of compounds possessing ethylene glycol units is presented in Scheme 1. Phenol **2** was prepared as follows. A mixture of 41 g (0.2 mol) tetraethylene glykol monomethyl ether **1** (TEG) and 64 g of dry pyridine was reacted with 41 g of tosyl chloride at −5 °C for 2 hours. Then the mixture was stirred at room temperature for 3 hours and poured into 250 g of ice with 100 mL of concentrated hydrochloric acid. The product was then extracted three times by benzene; the organic layer was washed with cold HCl, dried with potassium carbonate, filtered and evaporated. The yield was 40 g of a yellow viscous liquid of TEG-tosylate. ^1^H NMR (CDCl_3_, 300 MHz) for the intermediate product TEG-tosylate δ (ppm) 7.72 (d, 2H, ortho to SO_3_), 7.30 (d, 2H, ortho to CH_3_), 4.10 (t, 2H, CH_2_OAr), 3.65–3.45 (m, 14H, CH_2_O), 3.30 (s, 3H, CH_3_O), 2.38 (s, 3H, CH_3_Ar).

A mixture of 68 g (0.188 mol) of TEG–tosylate and 38 g (0.2 mol) of 4,4‘-biphenol was dissolved in 0.5 L ethanol/water (1:1) and heated under reflux. Sodium hydroxide (12 g) dissolved in 50 mL of water was added drop by drop over several hours. Boiling under reflux was continued for 4 days, the solution was then acidified with hydrochloric acid, ethanol was evaporated, and the aqueous mixture was left to stand in the refrigerator overnight. The crude substance was separated on fritted glass and crystallized from ethanol. Purification of crude phenol **2** was performed after its acetylation: 3 hours boiling with acetanhydride followed by cooling the reaction mixture on ice and further extraction of the product into dichloromethane. This protected compound was purified by column chromatography on silica gel using mobile phase dichloromethane/acetone (97:3), and the acetyl group was then removed by hydrolysis in a potassium hydroxide/ethanol mixture. Afterwards the solution was acidified by HCl, extracted by diethyl ether/acetone, dried by potassium sulfate and evaporated in vacuum. The yield was 23 g (0.06 mol, 30.5%). ^1^H NMR of the intermediate product **2** (DMSO, 300 MHz) δ (ppm) 7.50 and 7.41 (dd, 4H, ortho to –Ar), 6.98 (d, 2H, ortho to –OR), 6.81 (d, 2H, ortho to –OH), 4.11 (t, 2H, CH_2_OAr), 3.75 (t, 2H, CH_2_CH_2_OAr), 3.60–3.40 (m, 12H, CH_2_O), 3.22 (s, 3H, CH_3_).

Finally, **TL1** and **TL2** were obtained by conventional esterification with dicyclohexylcarbodiimide as condensation agent and dimethylaminopyridine as catalyst in tetrahydrofurane solution. ^1^H NMR of **TL1** (CDCl_3_, 300 MHz) δ (ppm) 8.18 (d, 2H, ortho to –COO), 7.60–7.40 (m, 4H, ortho to –Ar), 7.23 (d, 2H, ortho to –OCO), 6.98 (m, 4H, ortho to –OR), 4.18 (d, 2H, ArOCH_2_CH_2_O–), 4.05 (d, 2H, CH_2_OAr), 3.83 (d, 2H, ArOCH_2_C*H*_2_O), 3.80–3.50 (m, 12H, CH_2_O), 3.40 (s, 3H, OCH_3_), 1.90–1.20 (m, 20H, CH_2_), 0.90 (t, 3H, CH_3_).

^1^H NMR of **TL2** (CDCl_3_, 300 MHz) 8.18 (d, 2H, ortho to –OCO), 7.60–7.40 (m, 4H, ortho to –Ar), 7.23 (d, 2H, ortho to –OCO), 6.98 (m, 4H, ortho to –OR), 4.18 (d, 2H, ArOC*H*_2_CH_2_O–), 4.08 (m, 2H,CH_2_OAr), 3.83 (d, 2H, ArOCH_2_C*H*_2_O–), 3.80–3.50 (m, 12H, CH_2_O), 3.40 (s, 3H, OCH_3_), 1.90–1.30 (m, 3H, CH_2_CH), 0.90 (t + d, 6H, CH_3_). The optical rotation of **TL2** was determined in chloroform by using a polarimeter from Optical Activity Ltd. as [α]_D_^20^ +3.1 (*c* 0.15, CHCl_3_).

#### Preparation of **TL3**

**Scheme 2:**
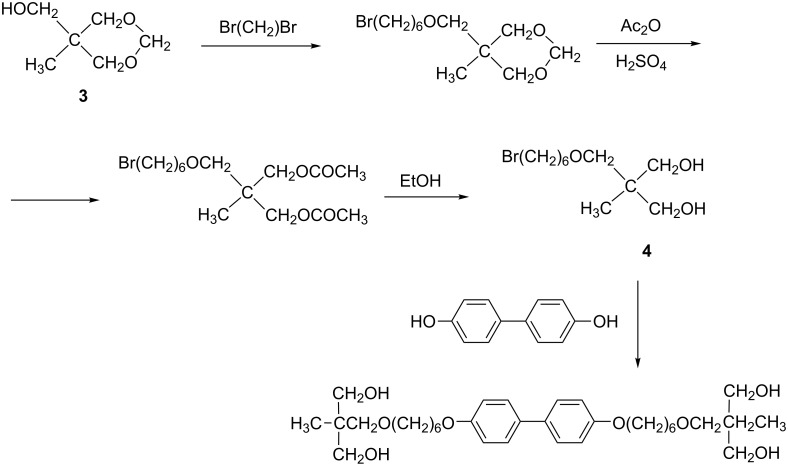
General procedure for synthesis of 2-(1-(6-(4'-(6-(3-hydroxy-2-(hydroxymethyl)-2-methylpropoxy)hexyloxy)biphenyl-4-yloxy)hexyloxy)methyl)-2-methylpropane-1,3-diol (**TL3**).

The synthetic procedure for the preparation of **TL3** with four hydroxy groups is presented in Scheme 2. Diol **4** was prepared from acetal **3** according to the procedure described previously [28]. ^1^H NMR (CDCl_3_, 300 MHz) for intermediate diol **4** δ (ppm) 3.70–3.50 (dd, 4H, CH_2_OH), 3.40 (m, 6H, CH_2_Br, CH_2_OCH_2_), 1.80 (quint., 2H, C*H*_2_CH_2_Br), 1.55 (quint., 2H, C*H*_2_CH_2_O), 1.40 (m, 4H, CH_2_), 0.80 (s, 3H, CH_3_).

The final compound 4,4´-biphenyl-bis(6-oxyhexyl-2,2-dimethylolpropyl ether) was prepared by the boiling of 10 g of 4,4´-biphenol with an excess of diol **4** in anhydrous potassium carbonate in acetone for 48 hours. The product was crystallized from acetone and purified by column chromatography on silica gel; the yield was 38%. ^1^H NMR (CDCl_3_, 300 MHz) for **TL3** δ (ppm) 7.50 (d, 4H, ortho to –Ar), 6.98 (d, 4H, ortho to –OR), 4.00 (t, 4H, CH_2_OAr), 1.80 (quint., 4H, C*H*_2_CH_2_OAr), 1.60–1.20 (m, 12H, CH_2_), 0.80 (s, 6H,CH_3_).

#### Preparation of **TL5**

**Scheme 3:**
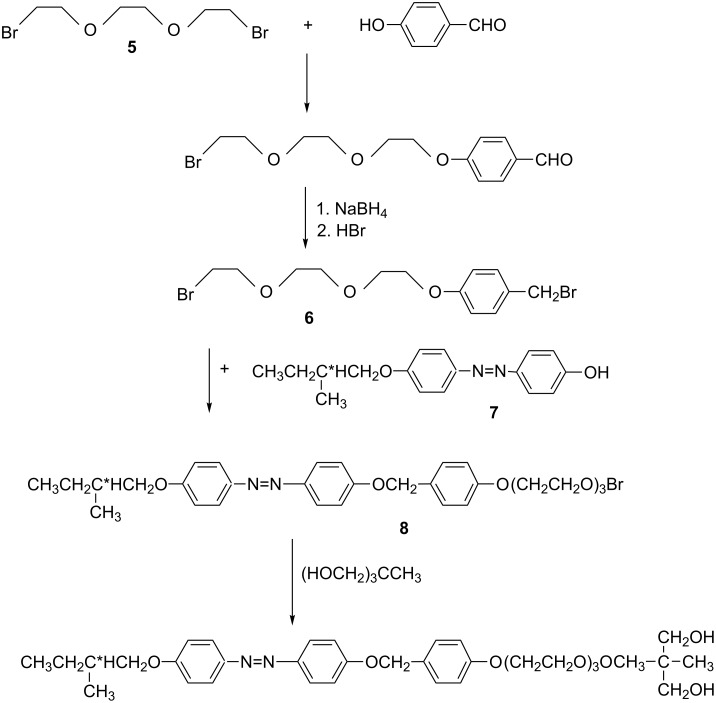
General procedure for the synthesis of chiral (*E*)-2-methyl-2-((2-(2-(2-(4-((4-((4-(2-methylbutoxy)phenyl)diazenyl)phenoxy)methyl)phenoxy)ethoxy)ethoxy)ethoxy)methyl)propane-1,3-diol (**TL5**).

The general procedure for the synthesis of chiral **TL5** possessing two hydroxy groups and ethylene glycol units is presented in Scheme 3. The bromide **6** was prepared from **5** by similar methods as those presented in [36]. ^1^H NMR of the intermediate product **6** (CDCl_3_, 300 MHz) δ (ppm) 7.30 (d, 2H, ortho to CH_2_Br), 6.88 (d, 2H, ortho to –O), 4.50 (s, 2H, ArCH_2_Br), 4.12 (t, 2H, CH_2_OAr), 3.90–3.60 (m, 8H, CH_2_O), 3.45 (t, 2H, CH_2_Br).

The mesogenic phenol **7** was prepared according to the synthetic procedure described in [36]. Intermediates **6** and **7** were reacted in equal molar ratio by boiling in acetonitrile in the presence of an excess of dry potassium carbonate for twenty hours. The reaction mixture was poured into water; the precipitate was filtered off and crystallized from acetone and from toluene. ^1^H NMR of intermediate product **8** (CDCl_3_, 300 MHz) δ (ppm) 7.86 (d, 4H, ortho to –N=N–), 7.39 (d, 2H, ortho to CH_2_), 7.10–6.90 (ddd, 6H, ortho to –O), 4.15 (t, 2H, ArOC*H*_2_CH_2_O), 3.90–3.60 (m, 10H, CH_2_O), 3.45 (t, 2H, CH_2_Br), 1.90, 1.60, 1.30 (m, 3H, CH_2_C*H), 1.02 (d, 3H, CH_3_), 0.97 (t, 3H, CH_3_).

Sodium hydride (0.1 mol) was slowly added to a solution of 0.2 mol of 1,1,1- tris(hydroxymethyl)ethane (pentaglycerine) in 0.25 L dry dimethylformamide. After one hour of stirring at room temperature (evolution of hydrogen), 0.05 mol of bromide **8** was added and the reaction mixture was kept at 50–60 °C overnight. Then the solvent was evaporated in vacuum and the solid residue was extracted several times with water to remove excess pentaglycerine. The resulting yellow residue was dried in vacuum and the crude product purified by column chromatography on silica gel using a mixture of chloroform and ethanol (90:10) as eluent. The yield was 7 g (0.011 mol, 22%).

^1^H NMR of **TL5** (CDCl_3_, 300 MHz) δ (ppm) 7.85 (d, 4H, ortho to –N=N–), 7.38 (d, 2H, ortho to –CH_2_), 7.10–6.90 (ddd, 6H, ortho to –O), 5.05 (s, 2H, Ar–CH_2_O), 4.15 (t, 2H, ArOCH_2_CH_2_O), 3.90–3.60 (m, 16H, CH_2_OCH_2_, CH_2_OH), 3.53 (s, 2H, C–CH_2_O), 1.90, 1.60, 1.30 (m, 3H, CH_2_C*H), 1.03 (d, 3H, CH_3_), 0.97 (t, 3H, CH_3_), 0.80 (s, 3H, C–CH_3_). The optical rotation of **TL5** was determined in chloroform, by using a polarimeter from Optical Activity Ltd., as [α]_D_^20^ +2.3 (*c* 0.05, CHCl_3_).

#### Thermotropic behaviour

Observation of the characteristic textures and their changes in POM was carried out on 12 μm thick glass cells, which were filled with LC material in the isotropic phase by means of capillary action. The inner surfaces of the glass plates are covered by indium-tin-oxide electrodes and polyimide layers unidirectionally rubbed, which ensures planar alignment of the molecules, e.g., bookshelf geometry in the smectic phase. In addition, for texture observation on samples with homeotropic alignment, free-standing films have also been used. For the preparation of a FSF, the liquid-crystalline material was mechanically spread over a circular hole (diameter 3 mm) in a metal plate. A LINKAM LTS E350 heating/cooling stage with a TMS 93 temperature programmer was used for temperature control, which enabled temperature stabilisation within ±0.1 K. Phase-transition temperatures and enthalpies were determined by differential scanning calorimetry (DSC - Pyris Diamond Perkin-Elmer 7) on samples of 3–6 mg hermetically sealed in aluminium pans in cooling/heating runs in a nitrogen atmosphere at a heating/cooling rate of 5 K min^−1^. The temperature was calibrated from extrapolated onsets of the melting points of water, indium and zinc. The enthalpy change was calibrated based on the enthalpies of melting of water, indium and zinc.

The small-angle X-ray scattering studies have been performed with Ni-filtered Cu Kα radiation (wavelength λ = 1.5418 Å). Small-angle scattering data from nonaligned samples (filled into Mark capillary tubes of 0.7 mm diameter) were obtained by using a Kratky compact camera (Aton Paar) equipped with a temperature controller and a one-dimensional electronic detector (M. Braun), the temperature being controlled within 0.1 K. For compounds possessing smectic phases, the layer thickness, *d*, was determined from Bragg’s law *n*λ = 2*d*sinθ, where *d* is calculated from the position of the small-angle (Θ = 0.2°–4.5°) diffraction peaks.
